# How many athletes are enough? Small-sample instability of reliability indices and the minimum sample size for a trustworthy minimal detectable change in two-trial sport field tests: a Monte Carlo study

**DOI:** 10.1186/s13102-026-01987-0

**Published:** 2026-08-13

**Authors:** İrfan Marangoz

**Affiliations:** Faculty of Sport Sciences, Kırşehir Ahi Evran University, Kırşehir, Türkiye

**Keywords:** Reliability, Intraclass correlation, Minimal detectable change, Measurement error, Sample size, Monte Carlo simulation, Sport field testing

## Abstract

**Background:**

Test-retest reliability of sport and rehabilitation field tests is usually reported from very small samples, yet how unstable the resulting indices are, and how many participants are needed, is rarely quantified. We used Monte Carlo simulation to evaluate relative (intraclass correlation coefficient, ICC) and absolute (minimal detectable change, MDC₉₅) reliability statistics under sport-realistic small-sample conditions.

**Methods:**

Two-trial data were simulated for a counter-movement-jump-like measure (mean 35 cm, SD 6 cm) in a fully crossed design: true ICC (0.60–0.90), sample size N (8-100) and a systematic between-trial shift (0–1 cm), 2000 replications per cell. For each dataset we computed ICC(2,1) and ICC(3,1) with 95% confidence intervals and absolute indices (standard error of measurement, typical error, coefficient of variation, MDC₉₅), evaluating bias, relative root-mean-square error (RMSE), interval coverage and qualitative-category instability, each with Monte Carlo standard errors. A trustworthy MDC was defined a priori as relative RMSE ≤ 15% (MDC within ~ 15% of truth; also 20%, 10%, 5%); robustness to heteroscedastic error was tested. Analyses used R.

**Results:**

ICC(2,1) confidence-interval coverage was nominal (~ 0.95), validating the design. The ICC(2,1) showed small-sample downward bias (up to -0.05 at *N* = 8, negligible by *N* = 100). The MDC₉₅ was far more volatile, its relative RMSE falling from ~ 26% (*N* = 8) to ~ 13% (*N* = 30) and ~ 7% (*N* = 100). The minimum N was ~ 15, ~30, ~ 75 and > 100 for the 20%, 15%, 10% and 5% tolerances. The qualitative ICC category was unstable in roughly half of small samples and, at the good/excellent boundary (0.90), remained ~ 50% unstable even at *N* = 100. A between-trial shift barely affected MDC instability but lowered the absolute-agreement ICC(2,1). The simulated MDC₉₅ curve matched its closed-form value; conclusions held under heteroscedastic error.

**Conclusions:**

For two-trial reliability studies resembling these conditions, about 30 participants were a practical minimum for a stable MDC₉₅ (15% tolerance), and more for stricter tolerances (model-specific, not universal). A high ICC did not guarantee a stable MDC; our findings suggest reporting absolute-error indices with their confidence intervals and sizing reliability studies around the precision of the MDC that drives individual decisions.

## Background

Reliable measurement is the foundation on which sport and rehabilitation field testing rests. Whether a practitioner is monitoring an athlete’s neuromuscular readiness with a counter-movement jump, tracking recovery of lower-limb power after injury, or deciding whether a training block has produced a real change, every inference depends on knowing how much of an observed difference reflects a genuine change in the individual and how much is measurement noise [1, 2]. Test-retest reliability studies are therefore not a statistical afterthought but a precondition for evidence-based practice: without a defensible estimate of measurement error, individual change scores cannot be interpreted, and group-level studies cannot specify the smallest worthwhile effect they are able to detect [3].

A recurring source of confusion is the distinction between relative and absolute reliability [1, 3]. Relative reliability - the intraclass correlation coefficient (ICC) and the Pearson correlation r - describes how well individuals keep their rank order across trials; being a ratio of between-subject to total variance, it is tied to the heterogeneity of the sample [3, 4]. Absolute reliability - the standard error of measurement (SEM), typical error of measurement (TEM), coefficient of variation (CV), minimal detectable change (MDC) and Bland-Altman limits of agreement (LoA) - describes error in the instrument’s original units and is, in principle, independent of that heterogeneity [2, 5, 6]. For decisions about individual athletes or patients the absolute indices, and especially the MDC, are what matter: the MDC₉₅ (commonly 1.96 × √2 × SEM) is the smallest single-person change that exceeds measurement noise with 95% confidence [3, 6]. This separation of “can we tell people apart” from “how large is the error” is well established [6], yet practice still leans on a single relative index, usually the ICC.

Two features of the applied literature make this reliance precarious. First, sport and exercise reliability studies are routinely conducted in very small samples: surveys report a median of roughly 19 participants, with many studies under-powered for the inferences they attempt [7], and dedicated test-retest investigations of field tests frequently enrol only 8–20 athletes. Second, a high ICC can coexist with poor absolute precision. Using data simulations, Warneke and colleagues showed that “excellent” ICC and r values (≥ 0.90) can accompany a mean absolute percentage error exceeding 20%, because correlation-based indices are insensitive to the systematic and random errors that determine whether an individual change is trustworthy [8]; the same concern has prompted editorial calls to report absolute error alongside reliability coefficients [9]. Compounding this, ICC point estimates are themselves small-sample sensitive: their sampling distribution is skewed and biased when N is small, and the qualitative “benchmark” categories derived from them (poor / moderate / good / excellent) [4] inherit that instability.

The statistical groundwork for sizing reliability studies is long established. Classic work derived sample-size requirements and optimal designs for estimating the ICC with adequate power or a confidence interval (CI) of desired width [10, 11, 12], and these methods underpin contemporary calculators. However, this literature was built almost entirely around the relative index: it answers “how many subjects do I need for a precise ICC?” rather than “how many subjects do I need before my MDC - the quantity practitioners actually act on - is trustworthy?” Recent sport-specific work has begun to probe the stability of reliability metrics across sample sizes in isokinetic strength testing [13]; this literature, however, has concentrated on the relative index and on describing how metrics fluctuate, rather than on the sample size at which the MDC itself becomes dependable. We build on this work to address that specific question.

Our work is closely related to, but methodologically distinct from, that recent study, which repeatedly subsampled a single dataset of 430 high-performance athletes to show how isokinetic-strength reliability metrics fluctuate as the analysed sample shrinks [13]. That study convincingly demonstrates that estimates become erratic at small N; however, because it resamples one real dataset, the underlying true reliability is unknown, so it can describe the scatter of estimates but cannot quantify their bias, their root-mean-square error (RMSE), or whether their confidence intervals attain nominal coverage. Sample-size effects on reliability estimation are, of course, well recognised - classical methods size studies for a precise ICC [10, 11, 12] and recent simulations show that reliability metrics scatter at small N [8, 13] - so our aim is not to rediscover this instability but to characterise it specifically for the MDC and to translate it into practical, tolerance-based sample-size guidance. Simulation-based tools have recently begun to recommend sample sizes for reliability and measurement-error studies [14]; our work is complementary to, but distinct from, these: it targets the MDC₉₅ that governs individual change decisions rather than the ICC/SEM confidence-interval width of a general clinimetric setting, validates the instability against an exact closed-form solution, quantifies qualitative-category misclassification, and tests robustness to heteroscedastic error under a sport-specific calibration. The present study is complementary by design: using a Monte Carlo model with a fully specified data-generating mechanism, the true ICC, SEM and MDC are known by construction, which allows bias, relative RMSE and CI coverage to be quantified against that known truth. We further (i) focus on the MDC, the index that governs individual change decisions, and translate an a priori accuracy tolerance into an explicit tolerance-to-sample-size decision table spanning several tolerance levels rather than reporting metric fluctuation descriptively; (ii) characterise the instability of the qualitative ICC category, including its persistence at the good/excellent boundary; (iii) test robustness to heteroscedastic error; and (iv) provide a transparent, reproducible, ADEMP-compliant framework whose conclusions are not tied to a single cohort or test modality. Our findings therefore corroborate the general message that small samples destabilise reliability estimates [8, 13] while adding the controlled, MDC-focused, decision-oriented quantification that a single-dataset resampling design cannot provide. We are explicit about what is, and is not, novel: for two trials the MDC relative-RMSE curve itself is analytically determined (a chi-square result independent of the true ICC, mean and SD), so we do not claim that descriptive curve as a discovery; rather, we validate it against its closed form and use it as the backbone of a sample-size decision table, while the genuinely simulation-dependent contributions are the finite-sample CI coverage, the qualitative-category instability, and the heteroscedastic-robustness results, none of which follow from the closed form or from single-dataset resampling.

We therefore conducted a Monte Carlo simulation, designed and reported according to the ADEMP framework [15], with a data-generating model calibrated to a counter-movement-jump-like field test (grand mean 35 cm, between-subject SD 6 cm). The study had three aims: (1) to verify that the simulation reproduces the nominal behaviour of ICC confidence intervals (a design check); (2) to quantify the small-sample bias of relative reliability indices and the instability - expressed as relative RMSE - of the MDC₉₅ across realistic sample sizes (*N* = 8, 15, 20, 30, 50, 75, 100), true ICC values (0.60, 0.80, 0.90) and a plausible between-trial shift (delta = 0–1 cm); and (3) to determine, for a range of pre-specified tolerance thresholds, the minimum sample size required for a trustworthy MDC and to evaluate how the qualitative ICC category behaves across sample sizes. We additionally tested the robustness of our conclusions to proportional (heteroscedastic) measurement error. On the basis of the differing sampling behaviour of variance ratios (the ICC) and variance components (the SEM and MDC), we hypothesised that the absolute index (MDC₉₅) would remain substantially more unstable than the relative index (ICC) across the small samples typical of field-test reliability studies, so that a dependable MDC would require a materially larger sample than a precise ICC.

## Methods

### Design and reporting

This is a Monte Carlo simulation study designed and reported following the ADEMP (Aims, Data-generating mechanism, Estimands, Methods, Performance measures) framework [15] and simulation-reporting guidance [16, 17]. The full protocol (aims, data-generating mechanism, estimands, methods, performance measures and number of replications) was specified a priori following the ADEMP framework and is provided with the deposited materials; the code, the random seed and the R session information are openly available in the Open Science Framework (OSF) repository (https://doi.org/10.17605/OSF.IO/PXQK3).

### Data-generating mechanism

Test-retest data with two trials were generated for a counter-movement-jump-like measure (grand mean µ = 35 cm; observed between-subject SD = 6 cm), with parameters anchored to published counter-movement-jump reliability data [18]. The counter-movement jump was chosen as the calibration because it is among the most widely used lower-limb field tests and its reliability parameters are well documented; the values used are representative of a continuous, approximately normal field-test outcome rather than specific to jump height, and because the primary instability metric (relative RMSE) is dimensionless the scale acts as a concrete calibration rather than a restriction (its transferability to other outcomes is examined in the Limitations). For participant i a true score was drawn and two trials were generated with independent normal error and an optional systematic between-trial shift δ:

$$\begin{aligned}\mathrm{T}_\mathrm{i}{^{\sim}}\;\mathrm{N}(\mu,\sigma_\mathrm{b}{^2}),\mathrm{X}_\mathrm{i1}&=\mathrm{T}_\mathrm{i}+\mathrm{e}_\mathrm{i1},\mathrm{X}_\mathrm{i2}=\mathrm{T}_\mathrm{i}+\delta\\&+\mathrm{e}_\mathrm{i2},\mathrm{e}^{\sim}\mathrm{N}(0,\sigma_\mathrm{w}{^2})\end{aligned}$$

The observed SD was held fixed and partitioned by the true ICC:

$$\begin{aligned}\sigma_\mathrm{b}=6\surd\mathrm{ICC},\;\sigma_\mathrm{w}&6\surd(1-\mathrm{ICC}),\;\mathrm{ICC}\\&=\sigma_\mathrm{b}{^2}/(\sigma_\mathrm{b}{^2}+\sigma_\mathrm{w}{^2})\end{aligned}$$

A fully crossed design varied true ICC (0.60, 0.80, 0.90), sample size N (8, 15, 20, 30, 50, 75, 100) and a systematic between-trial shift δ (0–1 cm). The 1 cm shift represents a small but realistic learning/fatigue effect for jump testing, of the order of the typical error of such measures [2, 18]. In a sensitivity analysis the error variance was instead made proportional to the true score (heteroscedastic error), scaled so that the population mean error variance equalled the within-subject variance, reflecting the proportional error common in sport performance data [1].

### Estimands and statistical estimators

The targets (‘truth’) were the true ICC, the true SEM = σ_w, the true TEM = σ_w, the true MDC₉₅, the true CV, and the true qualitative category (Koo & Li thresholds: <0.50 poor, 0.50–0.75 moderate, 0.75–0.90 good, ≥ 0.90 excellent) [4]. For each simulated dataset the following estimators were computed from a two-way analysis of variance (ANOVA; mean squares MSR, MSC, MSE):

$$\begin{aligned}\mathrm{SEM}&=\surd\mathrm{MSE},\;\mathrm{TEM}\\&=\mathrm{SD}_\mathrm{diff}/\surd{2},\;\mathrm{CV}\;(\%)\\&=(\mathrm{TEM}/\mathrm{mean})\times100,\mathrm{MDC}_{95}\\&=1.96\times\surd{2}\times\mathrm{SEM}\end{aligned}$$

Here SEM = √MSE captures within-subject (random) error only and is therefore insensitive to the systematic between-trial shift δ, which instead manifests in the absolute-agreement ICC(2,1) and the Bland-Altman bias. Because both the SEM (√MSE) and the TEM (SD_diff/√2) estimate the within-subject standard deviation σ_w in a two-trial homoscedastic design, their true values coincide; we nonetheless report both, as they are the conventions of, respectively, the clinimetric and the sport-science literatures and can diverge under heteroscedasticity or with more than two trials. Two ICC forms were used because they answer different questions: ICC(2,1), the two-way random-effects absolute-agreement coefficient, asks whether repeated scores agree in absolute terms and is therefore sensitive to a systematic between-trial shift; ICC(3,1), the two-way mixed-effects consistency coefficient, asks whether individuals keep their rank order and is largely insensitive to such a shift. A gap between the two therefore signals a systematic shift that practitioners should reconcile before quoting a single ‘reliability’ value. The ICC(2,1) 95% CI was computed by the exact procedure of McGraw & Wong [19] (which builds on the classical ICC framework of Shrout & Fleiss [20]), and the ICC(3,1) CI from the F distribution. Pearson r and Bland-Altman limits of agreement [5] were additionally computed as complementary descriptors (Pearson r is reported as a secondary relative index).

### Performance measures and the trustworthy-MDC criterion

Across 2000 replications per cell we computed, for each index, bias, empirical standard error (SE), RMSE, relative RMSE (%), 95% CI coverage, the qualitative-category instability rate (proportion of replications whose ICC category differed from the true category) and P(ICC ≥ 0.90); each is reported with its Monte Carlo standard error (MCSE: √(p(1 − p)/n_sim) for proportions, SD/√n_sim for means, and the delta-method value for relative RMSE) in all tables. The number of replications (2000) was chosen so that the MCSE of 95% coverage is about 0.5% (√(0.95·0.05/2000)); this is already an order of magnitude smaller than the between-condition differences of interest, so increasing the count to 5000 or 10000 would only shrink the MCSE to about 0.3% or 0.2% without altering any conclusion, and 2000 keeps the full factorial grid computationally light and exactly reproducible. As an internal check we also derived the closed-form value of the SEM/MDC relative RMSE, which for a two-trial design is 100·√(2 − 2·c₄(n − 1)) — where c₄ is the bias-correction constant and n − 1 the residual degrees of freedom — and is therefore invariant to the true ICC, mean and SD; the simulated estimate is reported alongside this analytic value. A trustworthy MDC was defined a priori as a relative RMSE no greater than 15%. We chose 15% as a pragmatic tolerance on three grounds: it keeps the sampling error of the estimated MDC (and hence of the individual change threshold it defines) below the magnitude of a typical smallest worthwhile change, so that whether an athlete is judged to have ‘really’ changed is unlikely to be reversed by the MDC’s own imprecision; it is of the same order as the sampling precision attainable for an estimated standard-deviation-type measurement-error statistic (the SEM, TEM or CV), whose relative RMSE is approximately 1/√(2ν) in the residual degrees of freedom ν, so that a 15% tolerance corresponds to roughly 22 residual degrees of freedom (about *N* = 23–24 for two trials; on the tested sample-size grid the smallest N meeting the tolerance is 30) - well within the reach of an adequately sized reliability study, for which the precision of the estimated SEM is itself a recognised, simulation-quantified design target [14]; and it was fixed a priori rather than after inspecting the estimates. Because no universal standard exists, we report the minimum N satisfying 20%, 10% and 5% as well, so that readers can apply their own tolerance. All analyses were performed in R 4.4.1 [21] using base R only (no add-on packages); ICCs were computed from ANOVA mean squares and their CIs as above. The full code and random seed are openly available in the OSF repository (see Availability of data and materials).

## Results

### Design validation: bias and confidence-interval coverage of the ICC

**Fig. 1:**
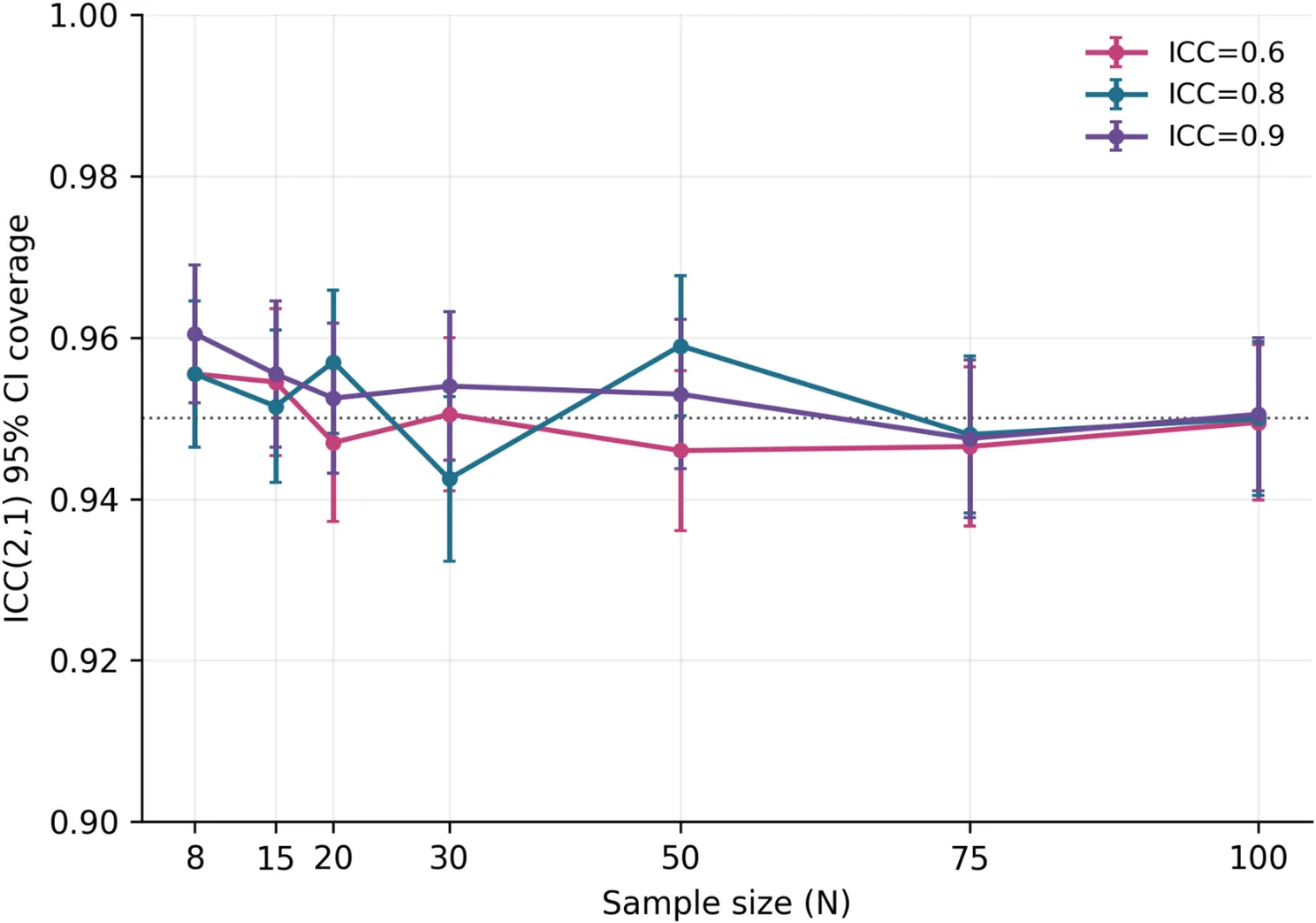
ICC(2,1) 95% confidence-interval coverage versus sample size. Empirical coverage of the nominal 95% confidence interval for the two-way random-effects absolute-agreement ICC, by true ICC (0.60, 0.80, 0.90) under no systematic shift. Error bars are Monte Carlo standard errors; the dotted line marks the nominal 0.95 target. Coverage at the nominal level validates the data-generating mechanism and interval procedure. ICC, intraclass correlation coefficient; N, sample size; CI, confidence interval

**Fig. 2:**
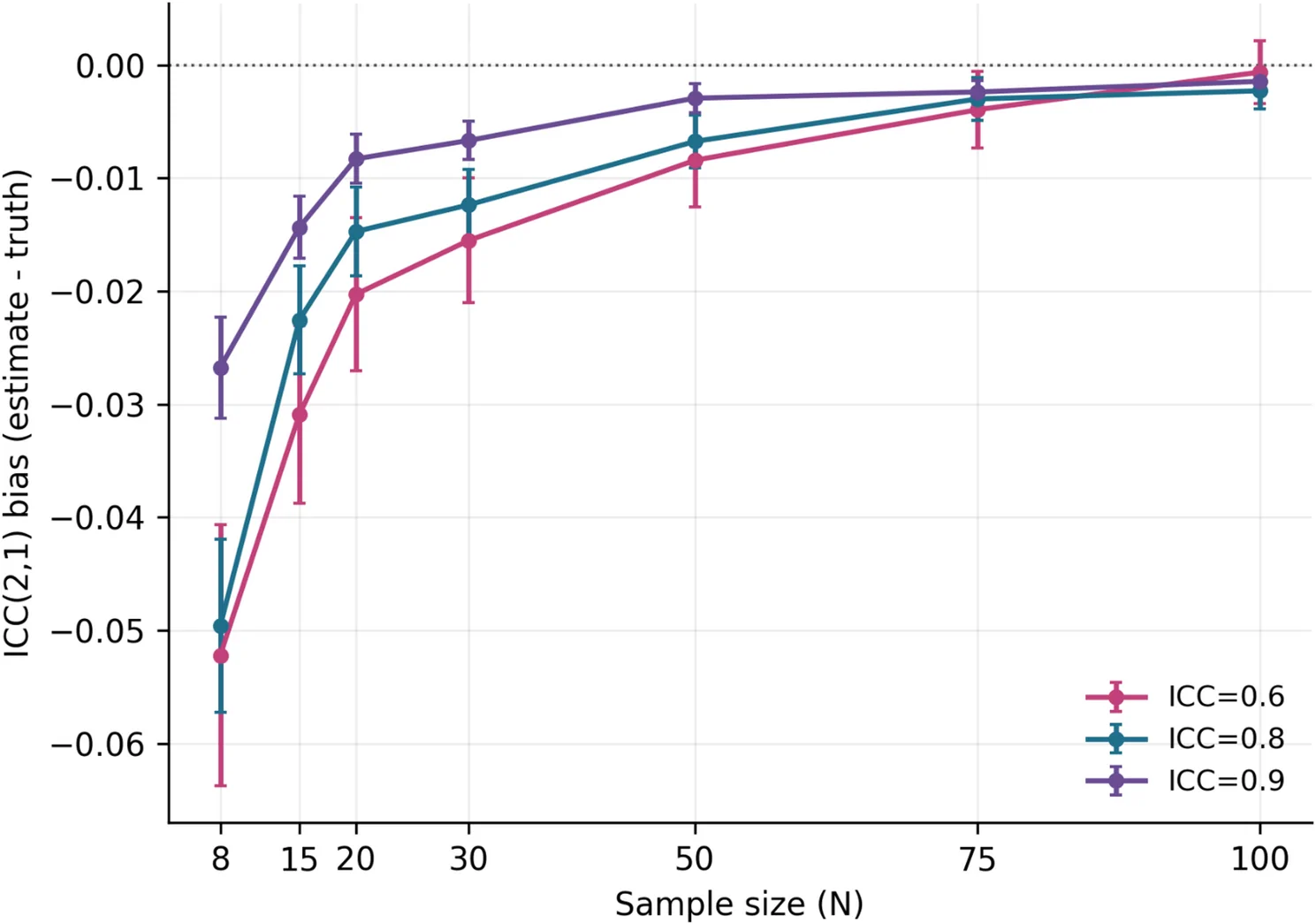
Small-sample bias of the ICC(2,1) versus sample size. Mean estimated minus true ICC across 2000 replications, by true ICC, with Monte Carlo standard-error bars; the dotted line marks zero bias. Estimates are biased downward at small N and approach zero by *N* = 100. ICC, intraclass correlation coefficient; N, sample size

ICC(2,1) 95% CI coverage was at the nominal level across all conditions (~ 0.94–0.96; Fig. 1), validating the estimator and interval implementation. ICC point estimates showed the expected small-sample downward bias (about − 0.05 at *N* = 8 for true ICC 0.60–0.80 and − 0.03 at ICC 0.90), shrinking to about − 0.001 by *N* = 100 (Fig. 2; Table 1).

**Table 1:** Performance of reliability indices by true ICC and sample size

True ICC	*N*	ICC mean (bias)	ICC 95% cov. (± MCSE)	MDC relRMSE % (± MCSE)	MDC relRMSE % analytic	ICC cat. instab. % (± MCSE)
0.6	8	0.548 (-0.052)	0.956 (± 0.005)	25.7 (± 0.37)	26.4	58 (± 1.1)
0.6	15	0.569 (-0.031)	0.955 (± 0.005)	18.7 (± 0.30)	18.8	44 (± 1.1)
0.6	20	0.580 (-0.020)	0.947 (± 0.005)	16.7 (± 0.25)	16.2	40 (± 1.1)
0.6	30	0.584 (-0.016)	0.951 (± 0.005)	13.5 (± 0.21)	13.1	31 (± 1.0)
0.6	50	0.592 (-0.008)	0.946 (± 0.005)	10.0 (± 0.15)	10.1	19 (± 0.9)
0.6	75	0.596 (-0.004)	0.947 (± 0.005)	8.4 (± 0.13)	8.2	11 (± 0.7)
0.6	100	0.599 (-0.001)	0.950 (± 0.005)	7.1 (± 0.12)	7.1	7 (± 0.6)
0.8	8	0.750 (-0.050)	0.956 (± 0.005)	26.1 (± 0.39)	26.4	53 (± 1.1)
0.8	15	0.777 (-0.023)	0.952 (± 0.005)	18.5 (± 0.29)	18.8	40 (± 1.1)
0.8	20	0.785 (-0.015)	0.957 (± 0.005)	15.9 (± 0.25)	16.2	33 (± 1.1)
0.8	30	0.788 (-0.012)	0.943 (± 0.005)	13.0 (± 0.22)	13.1	27 (± 1.0)
0.8	50	0.793 (-0.007)	0.959 (± 0.004)	10.1 (± 0.16)	10.1	20 (± 0.9)
0.8	75	0.797 (-0.003)	0.948 (± 0.005)	8.2 (± 0.13)	8.2	13 (± 0.8)
0.8	100	0.798 (-0.002)	0.950 (± 0.005)	7.0 (± 0.11)	7.1	10 (± 0.7)
0.9	8	0.873 (-0.027)	0.961 (± 0.004)	26.1 (± 0.41)	26.4	49 (± 1.1)
0.9	15	0.886 (-0.014)	0.956 (± 0.005)	18.9 (± 0.29)	18.8	50 (± 1.1)
0.9	20	0.892 (-0.008)	0.953 (± 0.005)	17.2 (± 0.26)	16.2	48 (± 1.1)
0.9	30	0.893 (-0.007)	0.954 (± 0.005)	13.2 (± 0.21)	13.1	51 (± 1.1)
0.9	50	0.897 (-0.003)	0.953 (± 0.005)	10.1 (± 0.15)	10.1	49 (± 1.1)
0.9	75	0.898 (-0.002)	0.948 (± 0.005)	8.3 (± 0.13)	8.2	50 (± 1.1)
0.9	100	0.899 (-0.001)	0.951 (± 0.005)	7.0 (± 0.11)	7.1	50 (± 1.1)

Homoscedastic error, no systematic shift; 2000 replications. Values are shown with their Monte Carlo standard error (MCSE). The ‘analytic’ column is the closed-form MDC relative RMSE, 100·√(2 − 2·c₄(*n* − 1)). Small fluctuations in the simulated MDC relative RMSE across true-ICC rows at a fixed N (for example 17.2% at ICC 0.90, *N* = 20 versus 15.9–16.7% at lower ICC and 16.2% analytic) reflect Monte Carlo sampling variability, not a dependence on the true ICC, which the theory rules out. The full estimator set (ICC(3,1), Pearson r, SEM, TEM, CV, Bland-Altman LoA) with MCSE is provided in the OSF repository (see Availability of data and materials).*ICC* intraclass correlation coefficient, *MDC* minimal detectable change, *RMSE* root-mean-square error

### Instability of the MDC₉₅ and minimum sample size

**Fig. 3:**
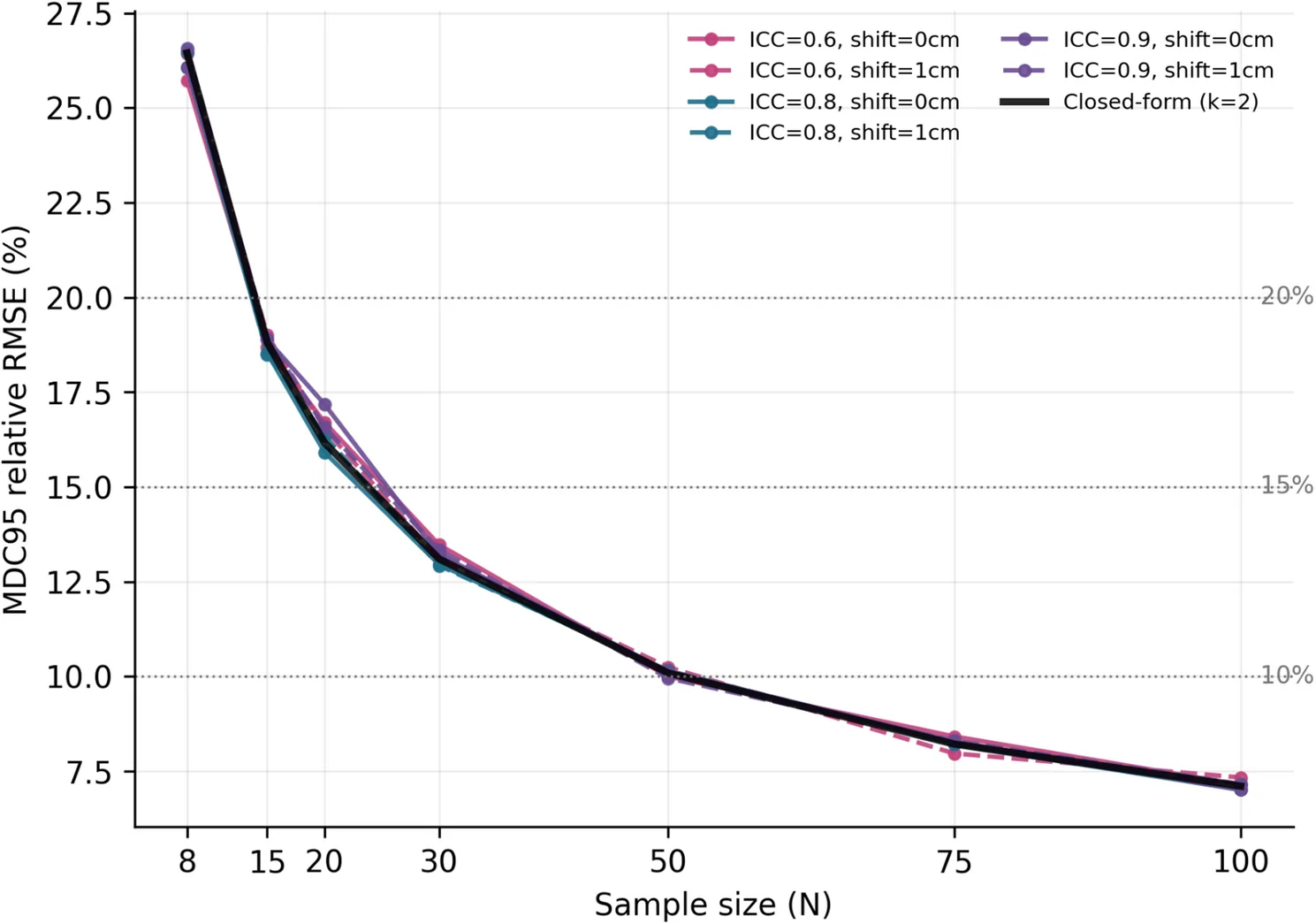
Relative root-mean-square error of the MDC₉₅ versus sample size. Relative RMSE (%) of the 95% minimal detectable change, by true ICC and between-trial shift (solid = 0 cm, dashed = 1 cm). The thick black line is the closed-form two-trial value 100·√(2 − 2·c₄(*n* − 1)); dotted horizontal lines mark the 10%, 15% and 20% tolerance thresholds. MDC, minimal detectable change; RMSE, root-mean-square error; ICC, intraclass correlation coefficient; N, sample size

The MDC₉₅ was markedly unstable in small samples: its relative RMSE fell from ~ 26% at *N* = 8 to ~ 19% (*N* = 15), ~ 16% (*N* = 20), ~ 13% (*N* = 30), ~ 10% (*N* = 50), ~ 8% (*N* = 75) and ~ 7% (*N* = 100) (Fig. 3; Table 1). The minimum sample size for a trustworthy MDC depended on the chosen tolerance (Table 2): about *N* = 15 for relative RMSE ≤ 20%, about *N* = 30 for ≤ 15%, about *N* = 75 for ≤ 10%, and more than 100 for ≤ 5%. The overall simulated requirement coincided with the exact closed-form value at every tolerance, confirming that the minimum N is governed by the residual degrees of freedom alone and is independent of the true ICC; individual per-ICC estimates can differ by a single grid step near a tolerance boundary (e.g. *N* = 50 versus 75 at the 10% line) purely from Monte Carlo noise within the MCSE, so the closed-form column is the invariance-bearing quantity. A systematic between-trial shift (delta = 1 cm) had little effect on MDC instability but, as expected, lowered the absolute-agreement ICC. The simulated values coincided with the closed-form baseline (Table 1, analytic column; Fig. 3), confirming that for two trials this curve is analytically determined and essentially invariant to the true ICC; the same dependence on residual degrees of freedom governed SEM, TEM and CV (all tabulated with MCSE in the OSF repository). All reported values carry their Monte Carlo standard errors (Table 1).

**Table 2:** Minimum sample size for a target MDC₉₅ stability

Target MDC95 stability (relative RMSE)	Minimum *N* (simulation)	Minimum *N* (closed-form)
≤ 20%	15	15
≤ 15%	30	30
≤ 10%	75	75
≤ 5%	> 100	> 100

Smallest N at which the MDC₉₅ relative RMSE meets each pre-specified tolerance, from the simulation (the largest requirement across all conditions: true ICC 0.60–0.90, shift 0–1 cm) and from the exact closed-form two-trial value 100·√(2 − 2·c₄(n − 1)). Their agreement confirms that the requirement is governed by the residual degrees of freedom alone and is independent of the true ICC. *MDC* minimal detectable change, *RMSE* root-mean-square error, *ICC* intraclass correlation coefficient

### Instability of the qualitative ICC category

**Fig. 4:**
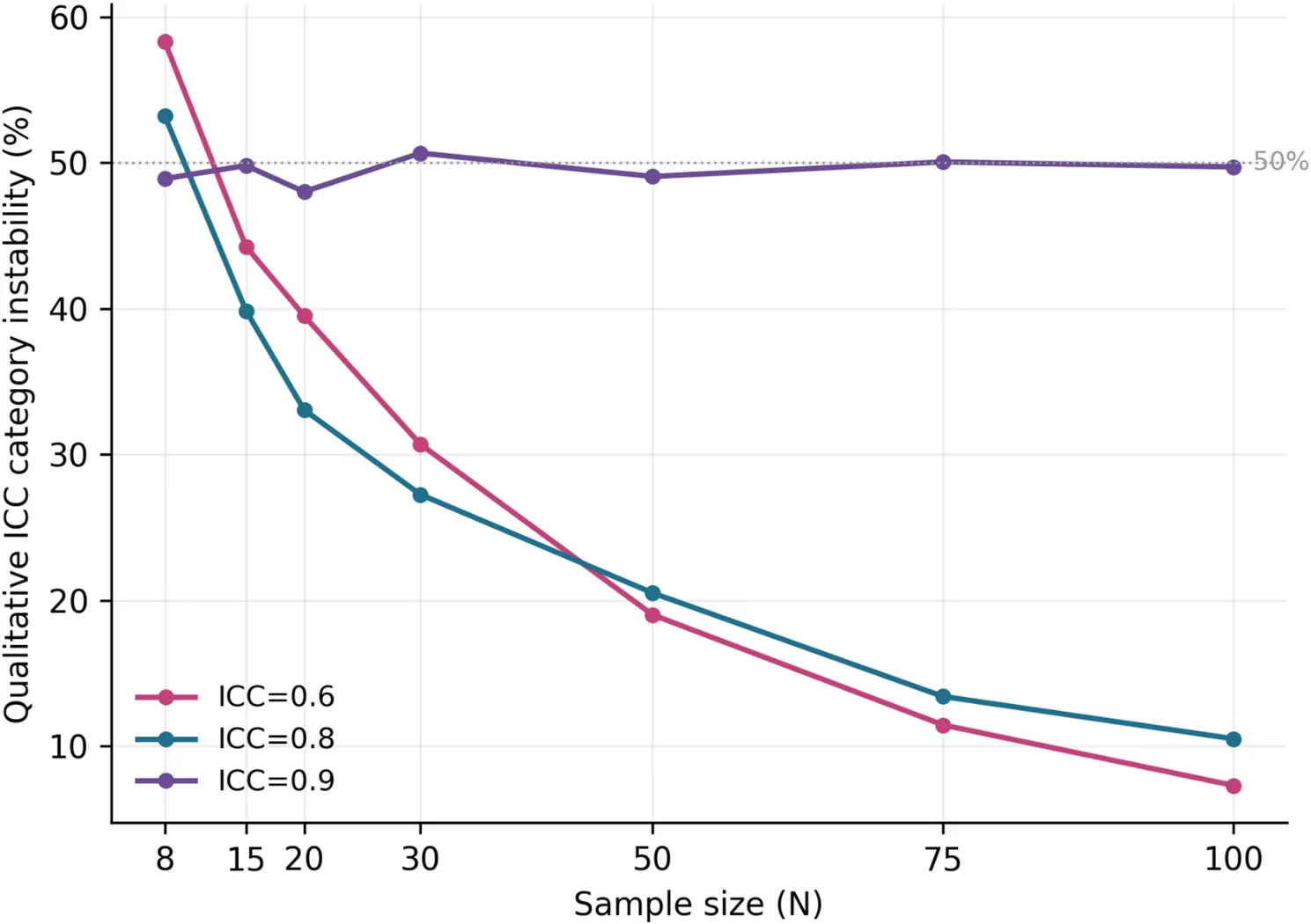
Instability of the qualitative ICC category versus sample size (no systematic shift). Proportion of replications whose ICC category (poor / moderate / good / excellent) differed from the true category, by true ICC, under no systematic between-trial shift. Instability stays near 50% (dotted line) at the good/excellent boundary (true ICC = 0.90) even at large N. ICC, intraclass correlation coefficient; N, sample size

In the no-shift condition, the qualitative ICC category was unstable in small samples, disagreeing with the true category in roughly half of *N* ≤ 15 samples (Fig. 4). When the true ICC lay on the good/excellent boundary (0.90), this instability remained about 50% even at *N* = 50 and *N* = 100, because estimates straddled the 0.90 cut-off; this reflects the fragility of hard cut-points applied to a sampling-variable statistic rather than a failure of estimation. Category instability is reported for the no-shift condition only: under a systematic between-trial shift the absolute-agreement ICC(2,1) is displaced by design, so the variance-defined qualitative category is no longer a well-posed estimand for this metric.

### Sensitivity to heteroscedastic error

**Fig. 5:**
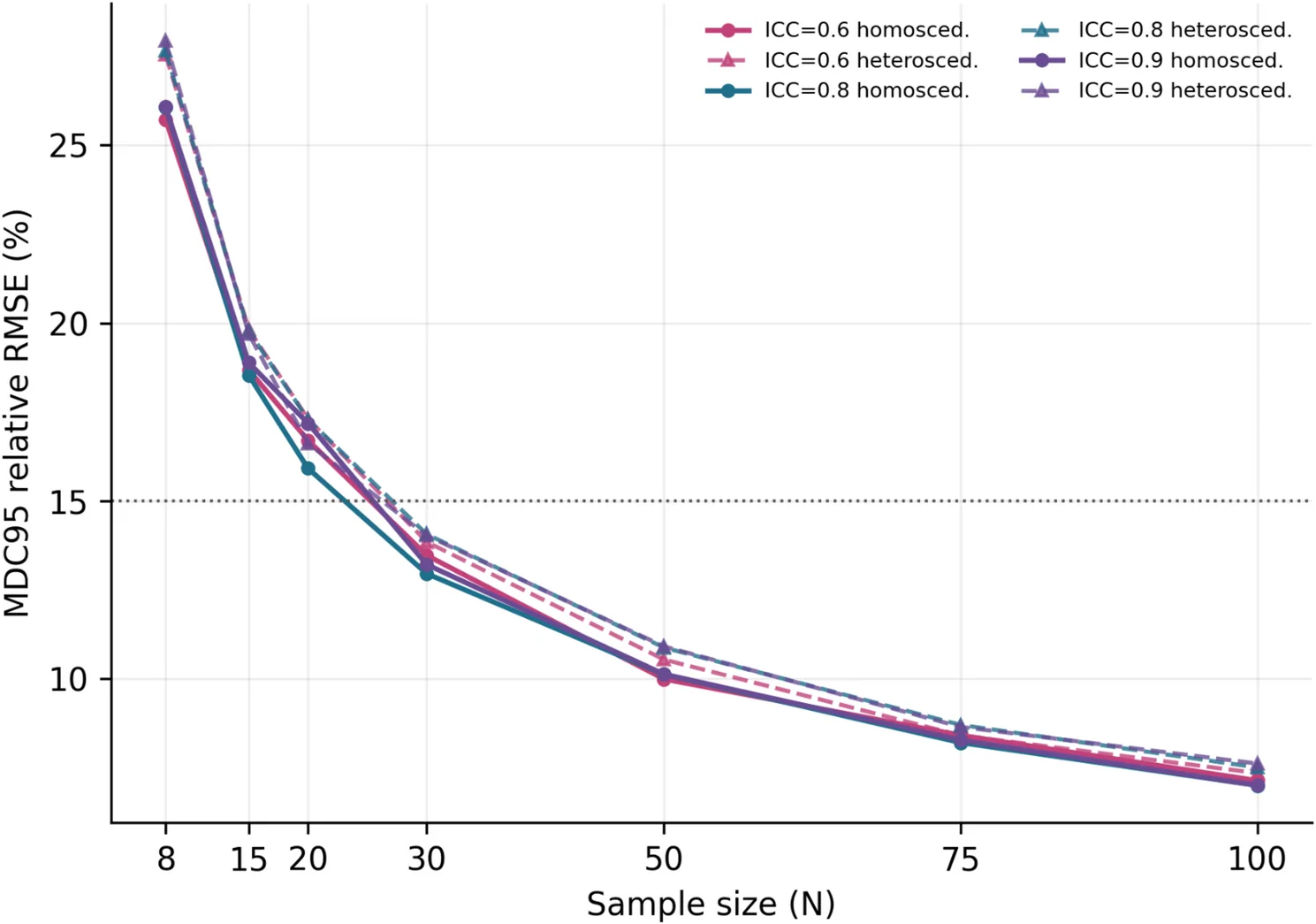
Sensitivity of MDC₉₅ instability to heteroscedastic error. Relative RMSE (%) of the MDC₉₅ under homoscedastic (solid) versus proportional / heteroscedastic (dashed) measurement error, by true ICC; the dotted line marks the 15% tolerance. Conclusions are unchanged. MDC, minimal detectable change; RMSE, root-mean-square error; ICC, intraclass correlation coefficient; N, sample size

Under proportional (heteroscedastic) error, MDC instability was marginally higher than in the homoscedastic case (relative RMSE about 27–28% at *N* = 8 and about 11% at *N* = 50) and ICC coverage was slightly reduced, but the N about 30 requirement for a 15% tolerance was unchanged (Fig. 5). The main conclusions are therefore robust to this realistic departure from the homoscedastic ideal.

### A practical sample-size summary

**Fig. 6:**
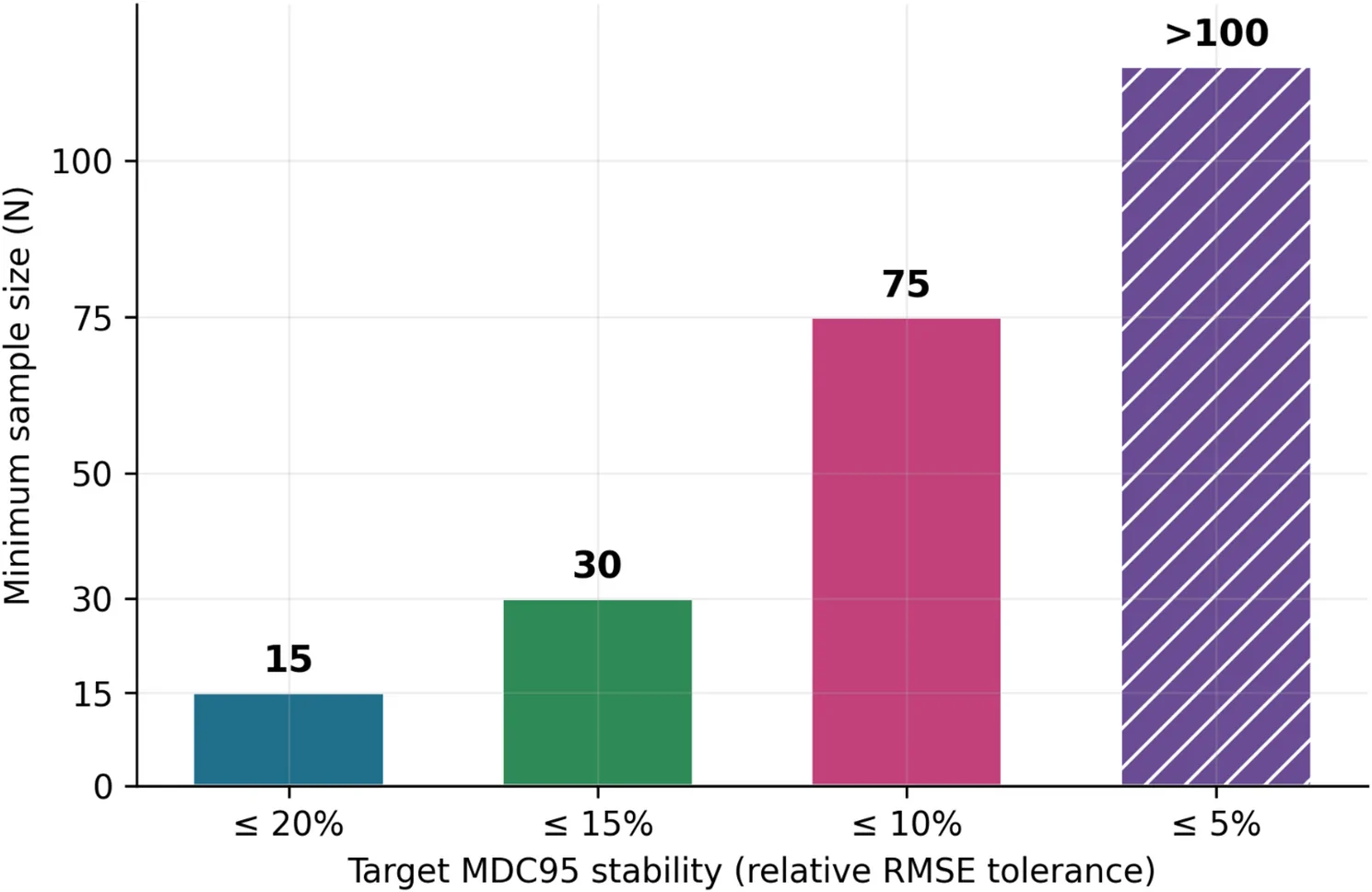
Minimum sample size for a trustworthy MDC₉₅ at each relative-RMSE tolerance. Bars show the smallest sample size at which the simulated (and closed-form) MDC₉₅ relative RMSE meets each pre-specified tolerance; the hatched bar (5%) was not reached even at *N* = 100 and is shown as > 100. The requirement is governed by the residual degrees of freedom and is therefore independent of the true ICC. MDC, minimal detectable change; RMSE, root-mean-square error; ICC, intraclass correlation coefficient; N, sample size

To make the sample-size requirements directly usable, Fig. 6 summarises, for each tolerance, the minimum sample size at which the MDC₉₅ becomes trustworthy: about 15 participants for a 20% relative-RMSE tolerance, about 30 for 15%, about 75 for 10%, and more than 100 for 5%. Because this requirement is fixed by the residual degrees of freedom (Table 2), the same values apply regardless of the true ICC; the figure is intended as a quick planning aid rather than a substitute for the full decision table.

## Discussion

This Monte Carlo study, calibrated to a counter-movement-jump-like field test and conducted under the ADEMP framework [15], quantified how small-sample instability degrades the reliability indices that sport and rehabilitation practitioners depend on. Five findings emerged.

### The design behaves as intended

Across all cells the 95% confidence interval for the ICC(2,1) achieved coverage close to the nominal 0.95, and the simulated MDC₉₅ relative-RMSE curve reproduced its closed-form value almost exactly (Table 1). This dual agreement - simulated coverage against the nominal 0.95, and the simulated MDC curve against its analytic solution - is more than a formality: it is strong evidence that the data-generating mechanism, estimands and interval procedures behave as analytic theory predicts [10, 11], so the findings below are properties of finite samples rather than artefacts of the simulation. Reporting such checks is precisely what the ADEMP framework encourages [15].

### Relative reliability is biased downward in small samples

ICC point estimates showed a small-sample downward bias of up to -0.05 at *N* = 8, shrinking to negligible levels by *N* = 50 and essentially vanishing by *N* = 100. The direction and magnitude are consistent with the known skew of the ICC sampling distribution [3, 12]. A bias of 0.05 may seem modest, but because it sits near the qualitative-category boundaries [4] its consequences are out of proportion to its size, as the fifth finding shows.

### Absolute precision is the binding constraint

The MDC₉₅ was far more volatile than the ICC. Its relative RMSE was approximately 26% at *N* = 8, 19% at *N* = 15, 16–17% at *N* = 20, 13% at *N* = 30, 10% at *N* = 50, 8% at *N* = 75 and 7% at *N* = 100. In other words, at the sample sizes most common in the field − 8 to 20 [7] - the MDC a practitioner reports can be wrong by a fifth to a quarter of its own value. Because the MDC is the quantity used to declare whether an individual has genuinely changed [3, 6], this instability translates directly into unreliable individual decisions. This operationalises, in the specific currency of the MDC, the broader warning that strong relative reliability does not guarantee acceptable absolute precision [8, 9]: the relative index (ICC) is already nearly unbiased at sample sizes where the absolute index (MDC) is still markedly unstable. As noted in the Introduction, for a two-trial design this MDC curve is analytically determined (it matched the closed-form value in Table 1); we therefore treat it as an analytically validated backbone for the decision table rather than as a novel empirical finding.

### Sample-size requirements depend on the tolerance chosen, with N about 30 a practical minimum

Rather than a single number, the minimum N depends on how much MDC instability one is willing to accept (Table 2). Using our a priori criterion of relative RMSE ≤ 15%, the MDC became trustworthy only at N about 30, across all true-ICC and shift conditions; a looser tolerance (≤ 20%) was met near N about 15, while a stricter one (≤ 10%) required N about 75 and ≤ 5% was not reached even at *N* = 100. These targets are larger than the samples typically used for field-test reliability work and complement, rather than contradict, the classical ICC-based sample-size guidance [10, 11, 12]: those methods size a study for a precise relative coefficient, whereas our criterion sizes it for a dependable absolute index. Because absolute precision governs individual change decisions, we suggest that, for two-trial field-test reliability studies resembling the simulated conditions, N about 30 is a reasonable practical minimum when the goal is to establish an MDC for practice. Figure 6 summarises this tolerance-to-sample-size mapping as a single decision aid, and we regard it as the central practical takeaway of the present work for applied researchers: decide how much MDC instability the intended decision can tolerate, then plan the study to the corresponding minimum sample size (about 15 for ≤ 20%, about 30 for ≤ 15%, about 75 for ≤ 10%, and more than 100 for ≤ 5%).

### The qualitative ICC category is unstable in small samples - and remains so at the 0.90 boundary

The qualitative rating [4] disagreed with the true category in roughly half of small samples. When the true ICC sat exactly on the good/excellent boundary (0.90), this category instability remained about 50% even at *N* = 50 and *N* = 100. We stress that this is not an estimator failure but the natural fragility of applying hard cut-points to a continuous, sampling-variable statistic: when the true value lies on a threshold, sampling variation moves the estimate across the cut-off essentially at random, and increasing N cannot remove it (a disagreement rate of about 50% is the expected value when the true coefficient sits exactly on a cut-point). The result reinforces long-standing cautions that the ICC is sample-dependent and should not be read as a fixed property of an instrument [1, 3], and it extends the critique of correlation-based indices [8] by showing that even the categorical interpretation of the ICC - not only its numerical adequacy - is untrustworthy near the benchmarks. The lesson is not that the ICC is useless, but that the ICC alone is insufficient: absolute indices (SEM, TEM, CV, MDC) should be reported with their confidence intervals, together with a Bland-Altman analysis of systematic and random error [2, 5, 6].

### The conclusions are robust to heteroscedastic error

When the error variance was made proportional to the true score (a common feature of sport performance data [1, 2]), the picture was essentially unchanged: MDC instability was marginally higher (for example, relative RMSE about 27–28% at *N* = 8 and about 11% at *N* = 50) and ICC coverage was slightly reduced, but the N about 30 recommendation for a ≤ 15% tolerance was unaffected. The main conclusions are therefore robust to this realistic departure from the homoscedastic ideal.

### Practical implications

Our findings suggest several practical points, which we frame as tendencies observed under the simulated model rather than as established rules. For researchers designing reliability studies, an MDC estimated from fewer than about 20 participants carries large sampling error - in our conditions a relative RMSE approaching a quarter of its own value - so planning around the precision of the absolute index that will drive decisions, and not only around ICC precision, would give the MDC its own sample-size justification. Figure 6 is intended to support precisely this planning step, since it displays the minimum sample size required at each tolerance in a single decision summary. For practitioners interpreting a published reliability study, a high ICC does not license confidence in absolute precision, so the SEM, TEM and MDC (with their confidence intervals) should be sought before an individual change is judged to be real. For editors and reviewers, requiring absolute-error indices with confidence intervals alongside the ICC, together with a Bland-Altman analysis of systematic and random error, would raise the evidentiary standard of reliability reporting. A more specific corollary follows from the shift results: because a systematic learning or fatigue effect between trials lowered the absolute-agreement ICC(2,1) but left the MDC essentially unchanged, when such an effect is plausible the choice between an agreement coefficient (ICC(2,1)) and a consistency coefficient (ICC(3,1)) may matter more for the reported reliability than the sample size.

### Limitations

Several constraints bound these conclusions, which should be read as applying under assumptions similar to those simulated. We modelled a single two-trial (k = 2) design; adding trials changes the precision of variance-component estimation and would shift the minimum-N targets [12], so the results should not be extrapolated to protocols with three or more repeated measurements. The systematic shift of 1 cm represents a small, realistic learning/fatigue effect for jump testing but does not span all plausible magnitudes. The main analysis assumed homoscedastic normal error; we tested proportional (heteroscedastic) error in a sensitivity analysis, but not log-normal, bounded, skewed, or outlier-contaminated outcomes, and departures from normality - common for time- and count-based field tests - could alter the finite-sample coverage and category-instability results, although the closed-form MDC curve, which depends only on the residual degrees of freedom, would be comparatively unaffected. The scale was fixed to a single counter-movement-jump-like calibration, chosen because it is among the most widely used field tests and its reliability parameters are well documented [18]; because relative RMSE is dimensionless, the MDC-instability curve is expected to transfer to other continuous, approximately normal two-trial outcomes with comparable signal-to-noise structure, but this transfer was not tested directly and requires empirical confirmation. We examined the absolute-agreement ICC(2,1) and consistency ICC(3,1); other ICC forms and agreement statistics may behave differently [4]. The 15% relative-RMSE criterion is a reasoned, pre-specified tolerance rather than a universal standard; we therefore report a range of thresholds (Table 2) so readers can apply their own. Finally, this is a simulation: it characterises estimator behaviour under a known model and does not, by itself, demonstrate the empirical superiority of any index or validate any particular field test.

### Future work

Priority extensions include designs with more than two trials; additional error structures (log-normal, bounded, skewed, outlier-contaminated); further ICC formulations and agreement statistics (for example ICC(1,1) and ICC(2,k), and the standardised typical error); calibrations spanning a wider range of signal-to-noise ratios and other sport and clinical field tests; and, importantly, an applied illustration in which the framework is used to plan and then audit a real field-test reliability study, so that the simulated guidance can be checked against empirical data.

## Conclusions

For sport and rehabilitation practitioners the single most actionable message is straightforward: report the absolute-error indices (SEM, TEM, MDC₉₅) with their confidence intervals rather than relying on the ICC alone, and size reliability studies around the precision of the absolute index that will drive individual change decisions. Under conditions designed to mimic sport and rehabilitation field testing, relative reliability (the ICC) stabilised earlier than that absolute precision. For two-trial reliability studies resembling the simulated conditions - a counter-movement-jump-like scale, approximately normal homoscedastic error, and true ICCs of 0.60–0.90 - a trustworthy MDC₉₅, defined a priori as a relative RMSE no greater than 15%, required approximately 30 participants, with larger samples for stricter tolerances (about 75 for 10%, more than 100 for 5%) and fewer for a looser 20% tolerance (about 15). Figure 6 condenses these targets into one decision summary and is, for applied researchers, the key practical message of this study. These sample sizes should be read as applying under the assumptions of the present simulation model rather than as universal requirements. A high ICC did not guarantee a stable MDC, and dichotomous ICC category labels were unsafe in small samples, remaining unstable at the good/excellent boundary even at *N* = 100. Our findings therefore suggest that both relative and absolute reliability indices - each reported with its confidence interval - deserve routine reporting, and that when an individual decision depends on the MDC its precision warrants its own sample-size justification.

## Data Availability

No empirical datasets were used; all data are simulated and fully reproducible from the deposited code. The complete R code (reliability_sim.R), the fixed random seed (20260614), the R session information (R 4.4.1) and the full per-cell estimator output are openly available in the Open Science Framework (OSF) at https://doi.org/10.17605/OSF.IO/PXQK3. Running reliability_sim.R reproduces every reported result without modification.

## Abbreviations

ADEMP: Aims, Data-generating mechanism, Estimands, Methods, Performance measures
ANOVA: Analysis of variance
CI: Confidence interval
CV: Coefficient of variation
ICC: Intraclass correlation coefficient
LoA: Limits of agreement
MCSE: Monte Carlo standard error
MDC: Minimal detectable change
MDC₉₅: Minimal detectable change at the 95% confidence level
RMSE: Root-mean-square error
SD: Standard deviation
SE: Standard error
SEM: Standard error of measurement
TEM: Typical error of measurement
